# Glioblastoma Multiforme Survivor With Radiation-Induced Consequences: A Case Report

**DOI:** 10.7759/cureus.29397

**Published:** 2022-09-21

**Authors:** Christina M Hunt, Virginia Thomas, Julia Alexander

**Affiliations:** 1 Orthopaedics, Alabama College of Osteopathic Medicine, Dothan, USA; 2 Medicine, Alabama College of Osteopathic Medicine, Dothan, USA; 3 Radiology, Alabama College of Osteopathic Medicine, Dothan, USA

**Keywords:** gbm, radiation induced, meningioma, glioblastoma, magnetic resonance imaging

## Abstract

Glioblastoma multiforme is an aggressive malignant tumor of the brain with a poor prognosis and no known cure. Current treatment options for this aggressive malignancy include surgical resection, adjuvant radiation therapy, and systemic pharmacological therapy. This case report presents one patient's experience with resolved glioblastoma multiforme treated by surgical resection and radiation therapy and discusses her later development and treatment of a radiation-induced meningioma. Despite developing radiation-induced morbidities, the patient experienced an extended life because of the aggressive treatment. It is thought that the young age of this patient at the time of diagnosis may have contributed to her prolonged survival time. When balancing aggressive treatment plans to increase survival time in glioblastoma multiforme patients, risks and potential consequences of treatment, such as post-surgical changes, vascular dementia, strokes, and meningiomas, should be weighed and discussed with the patient. Furthermore, striving for a high quality of life should be kept at the forefront of every treatment plan in all patients with glioblastoma multiforme.

## Introduction

Glioblastoma multiforme is an aggressive grade IV glioma with no known cure. Glioblastomas have an incidence rate of 3.20 per 100,000 population, are primarily diagnosed at a median age of 64 years, and depending on age and tumor histology, have a five-year survival rate of 5.5% [1]. The differential diagnosis for glioblastoma includes other brain tumors, metastasis, and neurological syndromes. Accurate diagnosis is made from the history, physical, imaging, biopsy, molecular testing, and overall clinical picture. Despite impressive improvements in diagnostic tools within the past three decades, treatment options for glioblastoma multiforme remain limited in scope. Surgical resection, adjuvant radiation therapy, and systemic pharmacological therapy remain the treatment's primary course of action [2]. Depending on the location, surgical removal and debulking may be limited. In the last half of the 20th century, it became clear that a potential side effect of radiation therapy includes the development of meningioma and other problems [3]. This case report presents the benefits of aggressive treatments of glioblastoma multiforme and discusses the risks and potentially associated morbidities of such treatments.

## Case presentation

A 54-year-old female presented to the hospital in 2009 with complaints of headaches and left-sided numbness for the past month. The patient reported no noticeable behavioral or vision changes, and she did not drink alcohol or smoke. She had no family history of tumors. The patient had a past medical history of a malignant glioblastoma grade IV that was treated with surgical resection and radiation in 1983. As a result of the resection surgery of the glioblastoma, the patient developed thalamic pain syndrome, now more commonly known as post-stroke pain. Other than her centralized pain, the patient remained symptom-free for 26 years.

**Figure 1:**
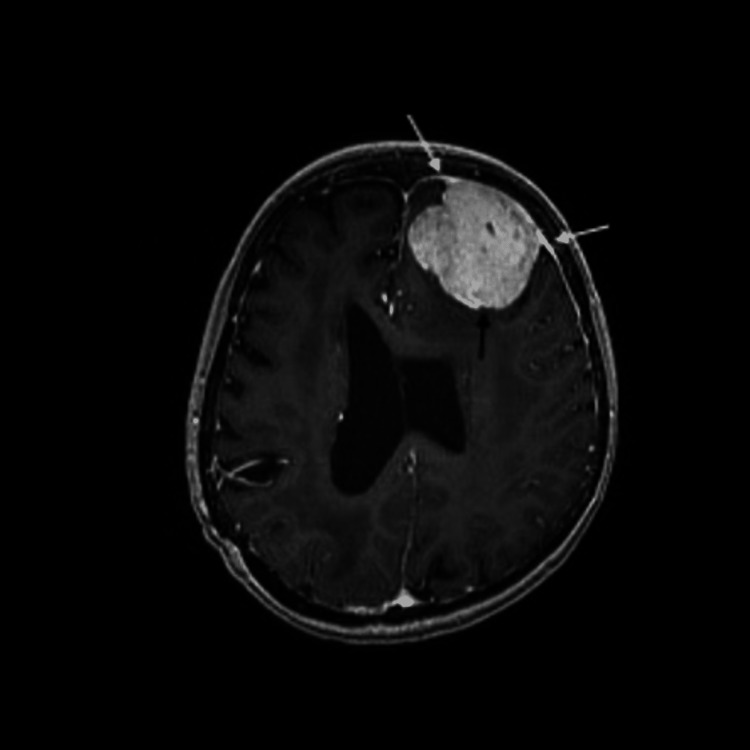
A T1 weighted post gadolinium axial MRI showing a 2.5cm, homogeneously enhancing meningioma (black arrow) with enhancing dural tails (white arrows).

**Figure 2:**
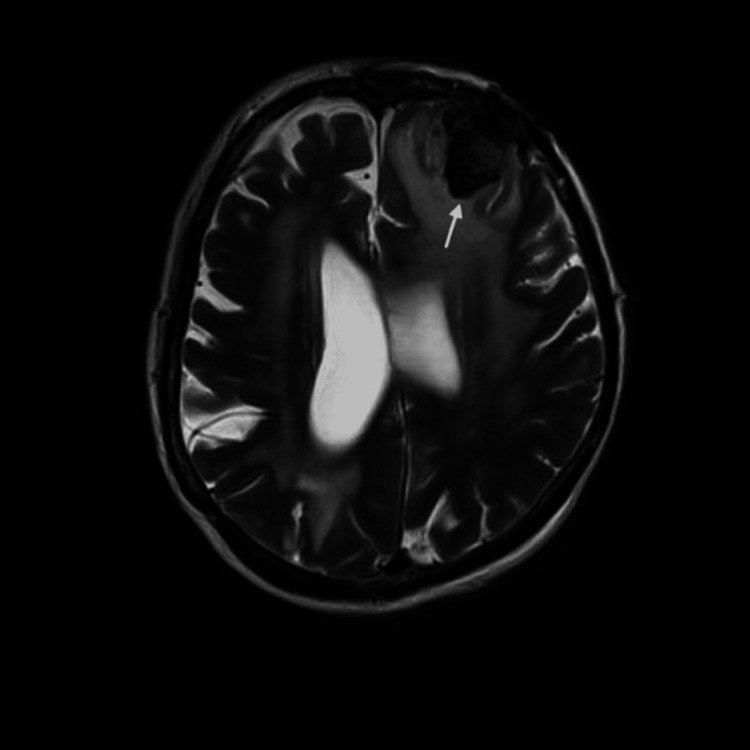
A T2 weighted axial MRI showing a 2.5cm meningioma (arrow) causing mass effect, with edema present.

**Figure 3:**
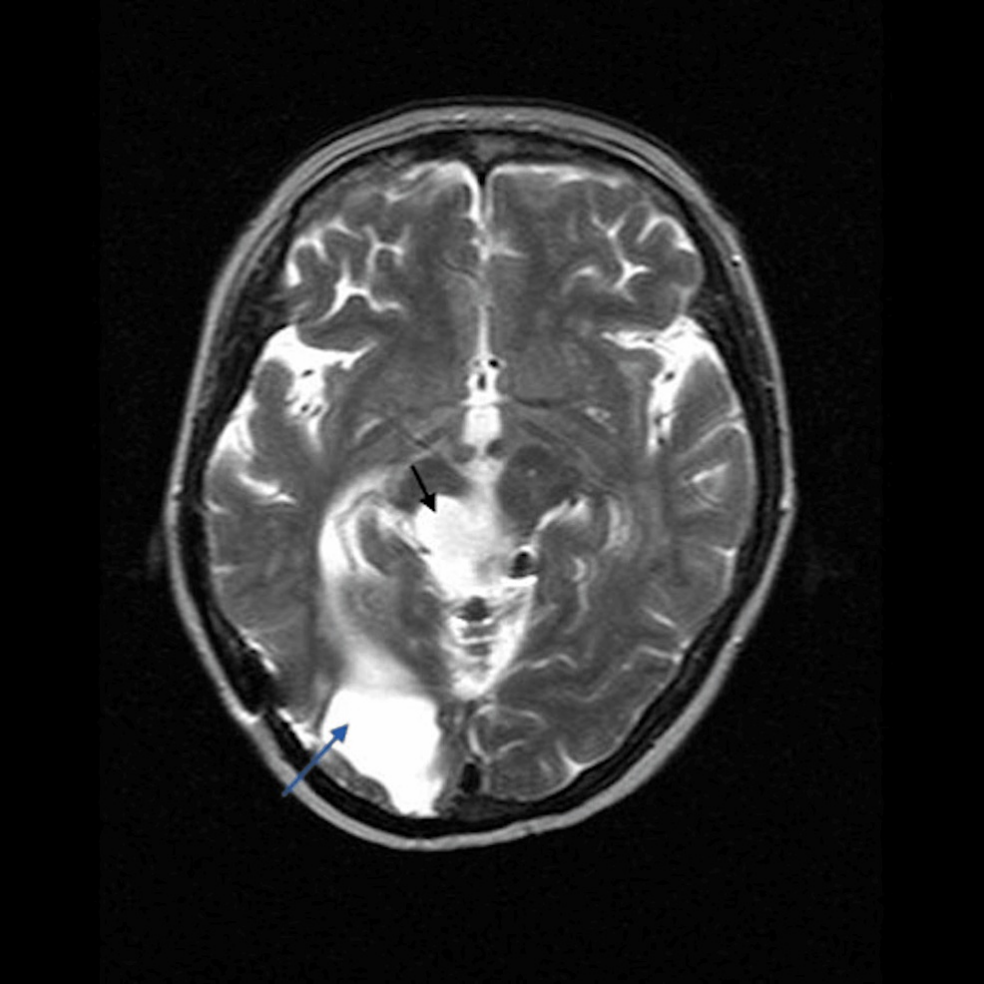
T2 weighted axial MRI showing cystic encephalomalacia in the right parietal/occipital region (grey arrow) in keeping with remote surgery for a glioblastoma, with additional post-surgical changes in the region of the midbrain (black arrow).

**Figure 4:**
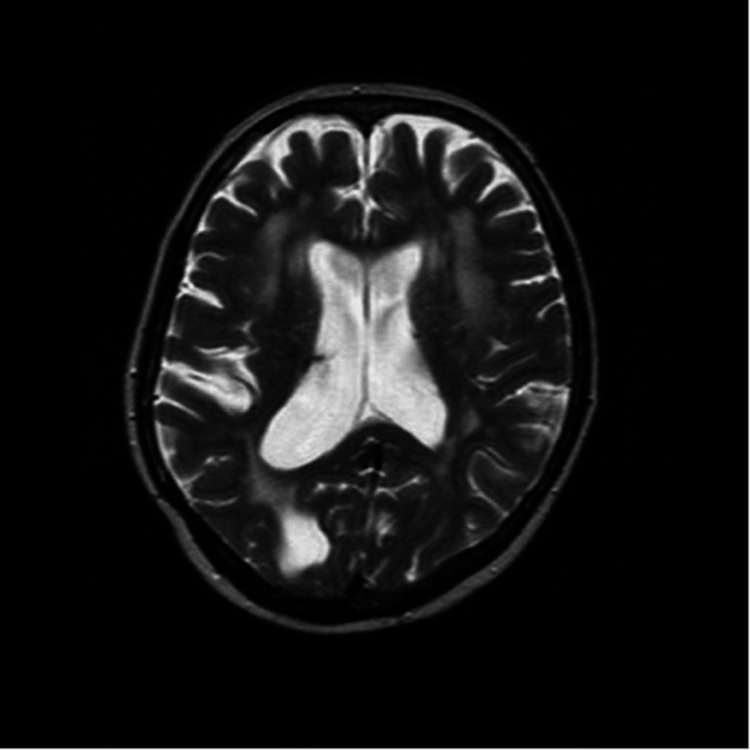
T2 weighted MRI showing ventriculomegaly and prominent sulci present in an ex-vacuo fashion, with extensive white matter disease throughout the supratentorial white matter related to previous glioblastoma treatment.

Given the patient's new neurological symptoms in 2009, a Magnetic Resonance Imaging (MRI) of the brain was performed, revealing a benign-appearing extra-axial lesion in the left frontal lobe (figure 1). This lesion exhibited homogenous enhancement, including an enhancing dural tail, associated mass effect, and vasogenic edema (figure 2). Imaging showed cystic encephalomalacia, ventriculomegaly, and white matter disease in keeping with post-treatment changes in 1983 (figure 3, 4). The patient's new lesion was felt to represent a benign meningioma that was subsequently treated with surgical resection. The following week the patient's headaches and left-sided numbness resolved.

Following the meningioma resection, the patient was followed. The patient remained symptom-free for a year but ultimately suffered a stroke in 2010, sparking noticeable mental decline. In 2013, the patient developed a subdural hematoma after a fall, requiring the patient to use a walker and become physically home-bound. The patient gradually had a further cognitive decline and became wheelchair-bound, requiring 24-hour supervision and assistance with basic activities of daily living and companionship. In 2017, the patient was further diagnosed with vascular dementia, depression, epilepsy, and sleep apnea. The patient was prone to urinary tract infections throughout her life, and she suffered two more strokes. She died in 2021 at age 65 after contracting the SARS-COV-2 virus, more than 38 years after her initial malignant glioblastoma diagnosis. Subjectively, the patient had an extensive network of support and exhibited an attitude of positivity, grace, and humility until her last moment.

## Discussion

Because the prognosis for glioblastoma multiforme is poor, this case report is significant secondary to the prolonged survival exhibited by the patient post diagnosis and treatment. This case benefits other glioblastoma multiforme patients and physicians treating such patients because extended life expectancy is possible. Although the patient lived 38 years post-diagnosis, the quality of life of those years should be discussed.

After surgical resection of the glioblastoma multiforme, the patient exhibited signs of thalamic pain syndrome, now more commonly known as post-stroke pain. The authors are unsure of the extent of the treatments the patient's previous providers tried for the patient's thalamic pain syndrome. The development of thalamic pain syndrome as a result of resection for glioblastoma multiforme, as seen in this patient, maybe one aspect to consider when weighing the risks of treatment.

In the last decade of her life, our patient developed vascular dementia, relied heavily on wheelchair usage after a fall, and was prone to urinary tract infections. This decreased quality of life was not seen until 26 years post radiation therapy. Radiation therapy may cause delayed side effects on neural tissue. These side effects include the potential development of benign or malignant neoplasms, a correlation that has been seen with the usage of high and low-dose radiation [3]. Endothelial damage of the blood-brain barrier after brain radiation can result in vasculopathies such as ischemic strokes and microbleeds occurring months to years after treatment [4]. Patients receiving brain radiation have also developed progressive dementia, ataxia, and urinary incontinence, causing severe disability [5]. The risk of developing radiation-induced neoplasms, vasculopathy, gait, and urinary disturbances should be discussed with patients before developing treatment plans for patients with glioblastoma multiforme.

This patient was diagnosed with glioblastoma multiforme at 28 years, much younger than most. Her diagnosis at a younger age may have contributed to the increased survival time exhibited by the patient. Survival estimates were slightly higher in younger patients diagnosed with glioblastoma multiforme; however, most patients with the disease are older [1]. Of note, the pharmacological options that exist today for glioblastoma multiforme, such as carmustine and temozolomide, were not available for this patient at the time of treatment but should be considered for current patients [2].

## Conclusions

When a patient is diagnosed with glioblastoma multiforme, the prognosis is poor. Aggressive treatment options such as surgical resection, adjuvant radiation therapy, and systemic pharmacological therapy may prove beneficial to prolong life expectancy but come with risks and consequences that can decrease quality of life. While this glioblastoma multiforme patient developed extensive secondary conditions such as thalamic pain syndrome, meningioma growth, vascular dementia, gait disturbance, urinary incontinence, and ischemic strokes from treatment with radiation and resection, it did allow a notable extension of good quality of life years.
